# Species conservation profile of moths (Insecta, Lepidoptera) from Azores, Portugal

**DOI:** 10.3897/BDJ.6.e23311

**Published:** 2018-04-19

**Authors:** Paulo A.V. Borges, Jose V. Pérez Santa-Rita, Rui Nunes, Anja Danielczak, Axel Hochkirch, Isabel R. Amorim, Lucas Lamelas-Lopez, Ole Karsholt, Virgílio Vieira

**Affiliations:** 1 cE3c – Centre for Ecology, Evolution and Environmental Changes / Azorean Biodiversity Group and Universidade dos Açores, Dep. de Ciências e Engenharia do Ambiente, Angra do Heroísmo, Azores, Portugal; 2 IUCN SSC Mid-Atlantic Islands Specialist Group, Angra do Heroísmo, Azores, Portugal; 3 Trier University, Department of Biogeography, D-54296 Trier, Germany; 4 Zoological Museum, Natural History Museum of Denmark, DK-2100, Copenhagen, Denmark; 5 cE3c – Centre for Ecology, Evolution and Environmental Changes / Azorean Biodiversity Group and Universidade dos Açores - Departamento de Biologia, Ponta Delgada, Azores, Portugal

**Keywords:** Azores, invasive species, islands, IUCN, Lepidoptera, moths, Portugal, species conservation profiles, rarity.

## Abstract

**Background:**

The few remnants of Azorean native forests harbour a unique set of endemic moths (Insecta, Lepidoptera), some of them under severe long term threats due to small sized habitats or climatic changes. In this contribution, we present the IUCN Red List profiles of 34 endemic moths to the Azorean archipelago, including species belonging to two diverse families: Noctuidae (11 species) and Crambidae (eight species). The objective of this paper is to assess all endemic Azorean moth species and advise on possible future research and conservation actions critical for the long-trem survival of the most endangered species.

**New information:**

Most species have a large distribution (i.e. 58% occur in at least four islands), very large extent of occurrence (EOO) and a relatively large area of occupancy (AOO). Only nine species are single-island endemics, three of them from Flores, three from São Miguel and one from Pico, São Jorge and Faial. Most of the species also experience continuing decline in habitat quality, number of locations and subpopulations caused by the ongoing threat from pasture intensification, forestry, invasive plant species and future climatic changes. The lack of new records may indicate that one of the species previously named is extinct (*Eupithecia ogilviata*). Therefore, we suggest as future conservation actions: (1) a long-term species monitoring plan and (2) control of invasive species.

## Introduction

Knowledge on Lepidoptera from the Azorean archipelago is still scarce and skewed towards the diurnal species of Rhopalocera (Borges et al. 2010). Most of the published work on Azorean Lepidoptera are species lists containing information such as locality, capture date, collectors and brief taxonomic annotations (Carvalho et al. 1999). The first taxonomic studies on Azorean Lepidoptera in Azores consisted of the description of new species only presenting a detailed description of the wing pattern (Warren 1905, Rebel 1940). Subsequently, new explorations of the insular entomofauna revealed new taxa, whose taxonomic descriptions were expanded with information about the morphology of genitalia and how to distinguish them from related species (Pinker 1971, Meyer et al. 1997, Nuss et al. 1997). The number of endemic species of Lepidoptera in the Azores has continued to increase in the last years. Recent studies focus not only on species description, but also on the ecology and distribution of new taxa, providing crucial information towards the conservation of these taxa (Wagner 2014, Wagner 2015a).

In this contribution, we present the IUCN Red List profiles of 34 moth species endemic to the Azores, including 11 owlet moths (Noctuidae), eight grass moths (Crambidae), three geometer moths (Geometridae), three Stathmopodidae, three ermine moths (Yponomeutidae), two snout moths or pyralid moths (Pyralidae), one twirler moth or gelechiid moth (Gelechiidae), one leaf-miner moth (Gracillaridae), one plume moth (Pterophoridae) and one fungus moth or tineid moth (Tineidae), which represent the majority of families present in the Azores (Borges et al. 2010).

Several of the endemic taxa here listed are known from a single collected individual, so that one of the sexes is unknown. The lack of reference collections for species identification and the low abundance of collected specimens for some taxa stresses the need for further studies that will allow a better understanding of the Lepidoptera fauna of the Azores.

The main objectives of this contribution are: 1) provide updated information on the distribution, abundance and ecology for the 34 Azorean endemic moths; 2) identification of the major threats involving these species; 3) the evaluation of the species conservation profiles for all known Azorean endemic moth species.

## Materials and Methods

To perform the IUCN Red List profiles, we followed the same procedure as in Borges et al. (2016), Borges et al. (2017) and Cardoso et al. (2017): i) the original species descriptions were investigated to learn about the habitats and ecology of the species; ii) recent literature was also consulted to obtain information about synonyms and critical information for the taxonomic notes; iii) for the calculation of AOO and EOO, we consulted the Azorean Biodiversity Portal and downloaded CSV files with the distribution of each species; iv) species images were obtained from specimens deposited in Coll. ZMUC (Credit: Anders Illum) and also from the repository available at the Azorean Biodiversity Portal, the most important source of information on Azorean biodiversity. Species distributions in the Azores were obtained from the list of Azorean biota (Borges et al. 2010) with the addition of recently described species (Wagner 2014, Wagner 2015a, Wagner 2015b, Wagner 2017).

Prior to the calculation of area of occupancy (AOO) and extent of occurrence (EOO), the 500 m × 500 m cells obtained from Azorean Biodiversity Portal were filtered to consider only the cells with high level of precision: 1 – very precise locality, usually with known UTM data; and 2 – literature locality not exceeding 25 km^2^. The centroid for each cell was calculated to obtain the distribution points for each species. The calculation of AOO and EOO was performed using the Geospatial Conservation Assessment Tool (GeoCAT) and using an approximation to the standard IUCN 2 km × 2 km cells (4 km^2^). Final maps with species distributions were produced using the IUCN standards with Google Earth (.kmz files).

Critical information on species threats and conservation were mostly obtained from Triantis et al. (2010) and Ferreira et al. (2016).

## Discussion

In this study we have analysed 34 species of moth described as endemic to the Azores, grouping the historical data and giving new information about their distribution, habitat, threats and proposals for their conservation. Twenty out of the 34 studied species are known from at least four islands and many of them are widely distributed within each island. These common species include all eight Crambidae, which are important pollinators of the Azorean native forest (Picanço et al. 2017).

We evaluated that 15 endemic species have an extent of occurrence (EOO) and area of occupancy (AOO) that is stable with a range of between 6,200-62,00 km^2^ for EOO and 44-584 km^2^ for AOO. Five species have a stable EOO but a decline in AOO decline, of which we must emphasise *Micrurapteryx bistrigella* (Rebel, 1940) and *Neomariania oecophorella* (Rebel, 1940) for presenting a low value of AOO. Five more species have an AOO and EOO decline, which present a low range between 1,950-8,900 km^2^ for EOO and 16-48 km^2^ for AOO. In addition to this, nine of these species have a very restricted distribution, occupying a unique island (three of them from Flores, another three from São Miguel, one from Faial, one from Pico and one from S. Jorge) and, therefore, they have a very small EOO and AOO. Amongst the analysed taxa, it should be noted that, for five species, only one individual is known (historical data), leaving one of the two sexes totally unknown. These species have low areas of occupation and are frequently restricted to a single patch of native forest. The lack of new records may indicate that one of the species previously named is extinct (*Eupithecia ogilviata*). In addition, many other species are in a critical conservation situation and actions should be taken with some urgency, namely the implementation of area-based management plans for those species distribution historical sites.

The recent description of four new endemic Noctuid moth species for the Azores (*Hadena azorica* Meyer & Fibiger, 2002; *Phlogophora kruegeri* Saldaitis & Ivinskis, 2006; *Apamea sphagnicola* Wagner, 2014; *Apamea ramonae* Wagner, 2015) challenges the notion that Lepidoptera are one of the most well studied taxonomic groups of insects in the Azores (cf. Borges et al. 2010, Borges et al. 2016b). Consequently, additional surveys are needed as well as taxonomic work on the earlier described species by Warren (1905) and Rebel (1940). There is also some urgency to perform standardised sampling of moths in the most important habitats of the Azores to investigate whether the negative impact of land-use changes observed for beetles and other Azorean arthropods (Cardoso et al. 2009) may also apply to this group.

**Figure 13.**
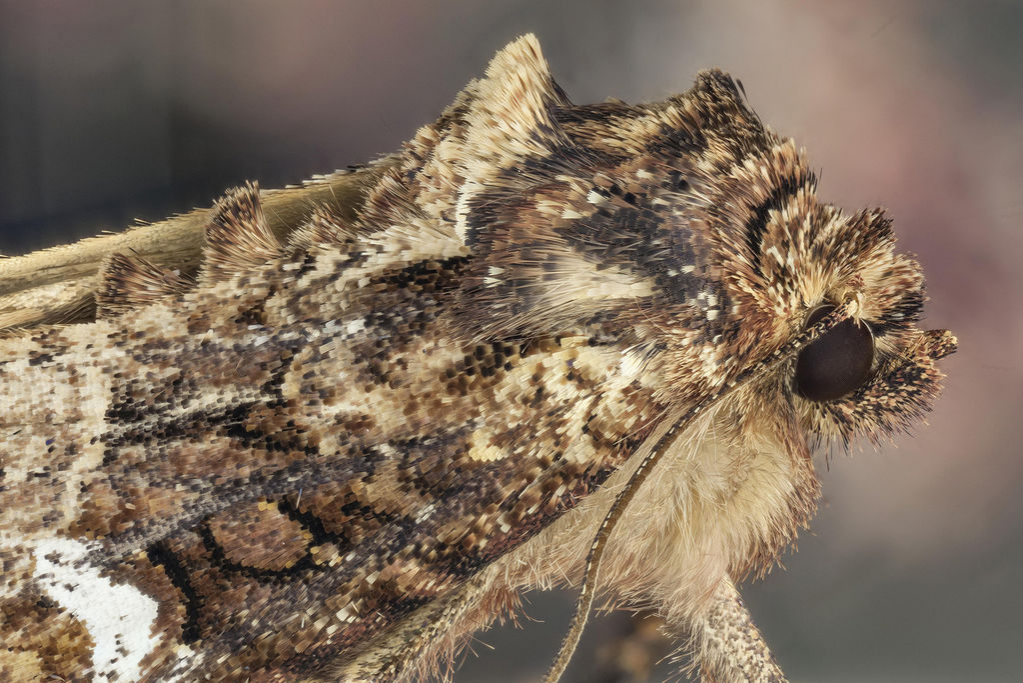
Extreme macro image of *Mesapamea storai* (Rebel, 1940) from Terra Brava (Terceira, Azores) (Credit: Javier Torrent).

Climate change is one of the prevailing threats across the world affecting numerous species and studies on some Azorean taxa show its negative effects, such as on Macaronesian bryophytes (Patiño et al. 2016) and Azorean spiders (Ferreira et al. 2016). Therefore and although the precise climate change impacts on Azorean moths are yet to be investigated, in a first approximation, we must assume a similar negative impact upon Lepidoptera. Further, most endemic moth species are now mostly restricted to the Azorean network of protected areas (RAA 2002) and their populations are decreasing due to pasture intensification, forestry (*Cryptomeria japonica* pulp plantations management) and invasive species (e.g. *Pittosporum undulatum*, *Hedychium gardnerianum*). Consequently, formal education and awareness is needed to allow future investments in habitat restoration of areas invaded by invasive plants or impacted by forestry and dairy-cow management, located mostly at mid elevations. The use of greatly magnified images (extreme macro photography) of Lepidoptera may be a successful strategy to inform the public about the ecological an aesthetical value of Azorean endemic moths (e.g. see Vieira 2006, Amorim et al. 2016, Arroz et al. 2016) (Fig. 13).

Concerning the most threathened Azorean moth species, a community monitoring plan is also crucial to generate data for the development of species recovery plans. Monitoring every ten years using the BALA protocol will inform about habitat quality (e.g. see Gaspar et al. 2011).

Discussion

## Supplementary Material

Supplementary material 1 *Eudonia interlinealis* map Data type: Map Google Earth
Brief description: Distribution of *Eudonia interlinealis* in the Azores islands.
File: oo_174738.kmz Anja Danielczak

Supplementary material 2 *Eudonia luteusalis* map Data type: Map Google Earth
Brief description: Distribution of *Eudonia luteosalis* in the Azores islands.
File: oo_174739.kmz Anja Danielczak

Supplementary material 3 *Eudonia melanographa* map Data type: Map Google Earth
Brief description: Distribution of Eudonia melanographa in the Azores islands.
File: oo_174740.kmz Anja Danielczak

Supplementary material 4 *Scoparia aequipennalis* map Data type: Map Google Earth
Brief description: Distribution of *Scoparia aequipennalis* in Azores islands.
File: oo_174741.kmz Anja Danielczak

Supplementary material 5 *Scoparia carvalhoi* map Data type: Map Google Earth
Brief description: Distribution of *Scoparia carvalhoi* in Azores islands.
File: oo_174742.kmz Anja Danielczak

Supplementary material 6 *Scoparia coecimaculalis* map Data type: Map Google Earth
Brief description: Distribution of *Scoparia coecimaculalis* in Azores islands.
File: oo_174743.kmz Anja Danielczak

Supplementary material 7 *Scoparia semiamplalis* map Data type: Map Google Earth
Brief description: Distribution of *Scoparia semiamplalis* in Azores islands.
File: oo_174744.kmz Anja Danielczak

Supplementary material 8 *Udea azorensis* map Data type: Map Google Earth
Brief description: Distribution of *Udea azorensis* in the Azores islands.
File: oo_174745.kmz Anja Danielczak

Supplementary material 9 *Brachmia infuscatella* map Data type: Map Google Earth
Brief description: Distribution *Brachmia infuscatella* in Azores islands.
File: oo_174746.kmz Anja Danielczak

Supplementary material 10 *Cyclophora azorensis* map Data type: Map Google Earth
Brief description: Distribution of *Cyclophora azorensis* in the Azores islands.
File: oo_174747.kmz Anja Danielczak

Supplementary material 11 *Eupithecia ogilviata* map Data type: Map Google Earth
Brief description: Distribution of *Eupithecia ogilviata* in Faial island.
File: oo_174748.kmz Anja Danielczak

Supplementary material 12 *Xanthorhoe inaequata* map Data type: Map Google Earth
Brief description: Distribution of *Xanthorhoe inaequata* in the Azores islands.
File: oo_174749.kmz Anja Danielczak

Supplementary material 13 *Micrurapteryx bistrigella* map Data type: Map Google Earth
Brief description: Distribution of *Micrurapteryx bistrigella* in Azores islands.
File: oo_174750.kmz Anja Danielczak

Supplementary material 14 *Apamea ramonae* map Data type: Map Google Earth
Brief description: Distribution of *Apamea ramonae* in Flores island.
File: oo_174751.kmz Anja Danielczak

Supplementary material 15 *Apamea sphagnicola* map Data type: Map Google Earth
Brief description: Distribution of *Apamea sphagnicola* in Azores islands.
File: oo_174752.kmz Anja Danielczak

Supplementary material 16 *Hadena azorica* map Data type: Map Google Earth
Brief description: Distribution of *Hadena azorica* in São Jorge island.
File: oo_174754.kmz Anja Danielczak

Supplementary material 17 *Melanchra granti* map Data type: Map Google Earth
Brief description: Distribution of *Melanchra granti* in Azores islands.
File: oo_174773.kmz Anja Danielczak

Supplementary material 18 *Mesapamea storai* map Data type: Map Google Earth
Brief description: Distribution of *Mesapamea storai* in Azores islands.
File: oo_174755.kmz Anja Danielczak

Supplementary material 19 *Noctua atlantica* map Data type: Map Google Earth
Brief description: Distribution of *Noctua atlantica* in Azores islands.
File: oo_174756.kmz Anja Danielczak

Supplementary material 20 *Noctua carvalhoi* map Data type: Map Google Earth
Brief description: Distribution of *Noctua carvalhoi* in Azores islands.
File: oo_174757.kmz Anja Danielczak

Supplementary material 21 *Phlogophora cabrali* map Data type: Map Google Earth
Brief description: Distribution of *Phlogophora cabrali* in Azores islands.
File: oo_174758.kmz Anja Danielczak

Supplementary material 22 *Phlogophora furnasi* map Data type: Map Google Earth
Brief description: Distribution of *Phlogophora furnasi* in Azores islands.
File: oo_174759.kmz Anja Danielczak

Supplementary material 23 *Phlogophora interrupta* map Data type: Map Google Earth
Brief description: Distribution of *Phlogophora interrupta* in Azores islands.
File: oo_174760.kmz Anja Danielczak

Supplementary material 24 *Phlogophora kruegeri* map Data type: Map Google Earth
Brief description: Distribution of *Phlogophora kruegeri* in Flores island.
File: oo_174761.kmz Anja Danielczak

Supplementary material 25 *Stenoptilia meyeri* map Data type: Map Google Earth
Brief description: Distribution of *Stenoptilia meyeri* in São Miguel island.
File: oo_174762.kmz Anja Danielczak

Supplementary material 26 *Homoeosoma miguelensis* map Data type: Map Google Earth
Brief description: Distribution of *Homoeosoma miguelensis* in São Miguel island.
File: oo_174763.kmz Anja Danielczak

Supplementary material 27 *Homoeosoma picoensis* map Data type: Map Google Earth
Brief description: Distribution of *Homoeosoma picoensis* in Pico island.
File: oo_174764.kmz Anja Danielczak

Supplementary material 28 *Neomariania incertella* map Data type: Map Google Earth
Brief description: Distribution of *Neomariania incertella* in Flores island.
File: oo_174765.kmz Anja Danielczak

Supplementary material 29 *Neomariania oecophorella* map Data type: Map Google Earth
Brief description: Distribution of *Neomariania oecophorella* in Azores islands.
File: oo_174766.kmz Anja Danielczak

Supplementary material 30 *Neomariania scriptella* map Data type: Map Google Earth
Brief description: Distribution of *Neomariania scriptella* in Azores islands.
File: oo_174767.kmz Anja Danielczak

Supplementary material 31 *Eudarcia atlantica* map Data type: Map Google Earth
Brief description: Distribution of *Eudarcia atlantica* in Azores islands.
File: oo_174768.kmz Anja Danielczak

Supplementary material 32 *Argyresthia atlanticella* map Data type: Map Google Earth
Brief description: Distribution of *Argyresthia atlanticella* in Azores islands.
File: oo_174771.kmz Anja Danielczak

Supplementary material 33 *Argyresthia minusculella* map Data type: Map Google Earth
Brief description: Distribution of *Argyresthia minusculella* in Azores islands.
File: oo_174772.kmz Anja Danielczak

Supplementary material 34 *Argyresthia poecilella* map Data type: Map Google Earth
Brief description: Distribution of *Argyresthia poecilella* in São Miguel island.
File: oo_174769.kmz Anja Danielczak

## Figures and Tables

**Figure 1.**
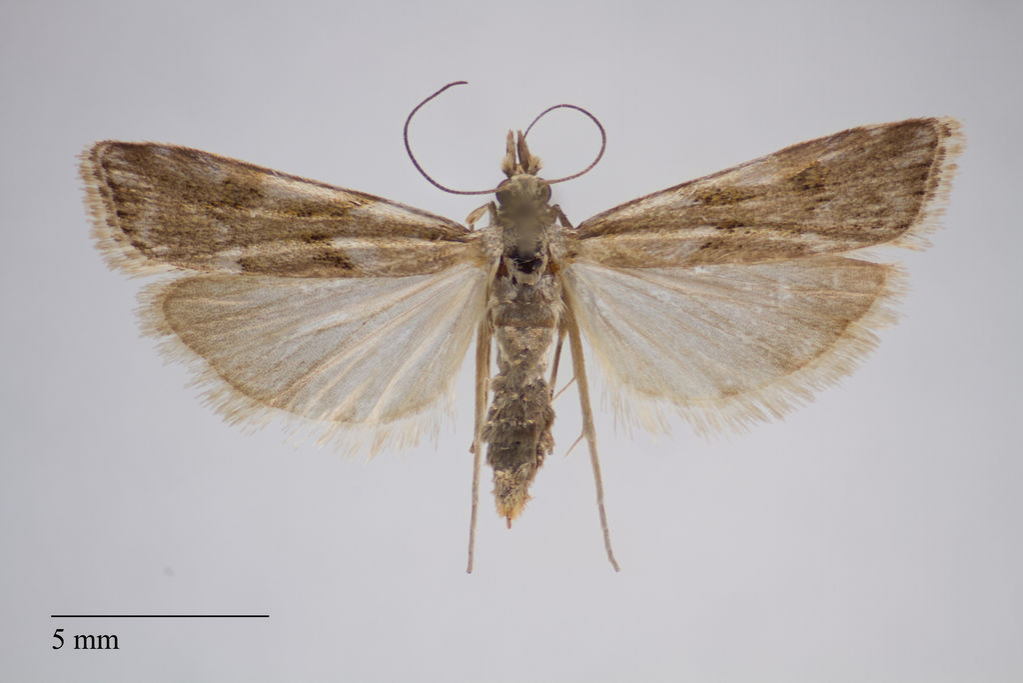
*Eudonia interlinealis* (Warren, 1905) from Pico (Azores, Portugal) deposited in Coll. ZMUC (Credit: Anders Illum).

**Figure 2.**
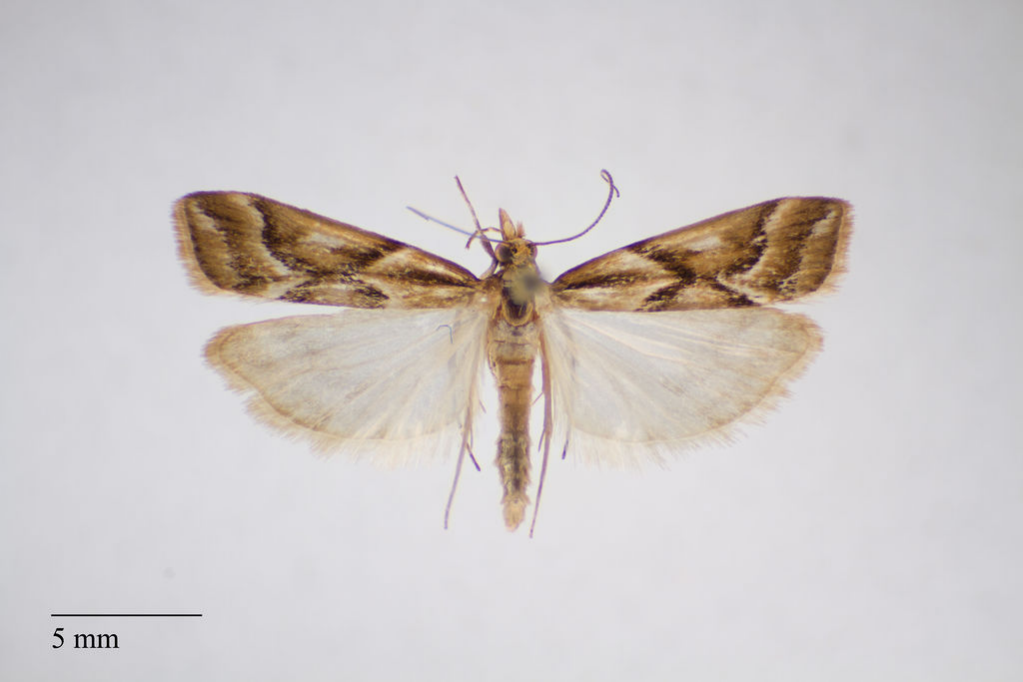
*Eudonia luteosalis* (Hampson, 1907) from Pico (Azores, Portugal) deposited in Coll. ZMUC (Credit: Anders Illum).

**Figure 3.**
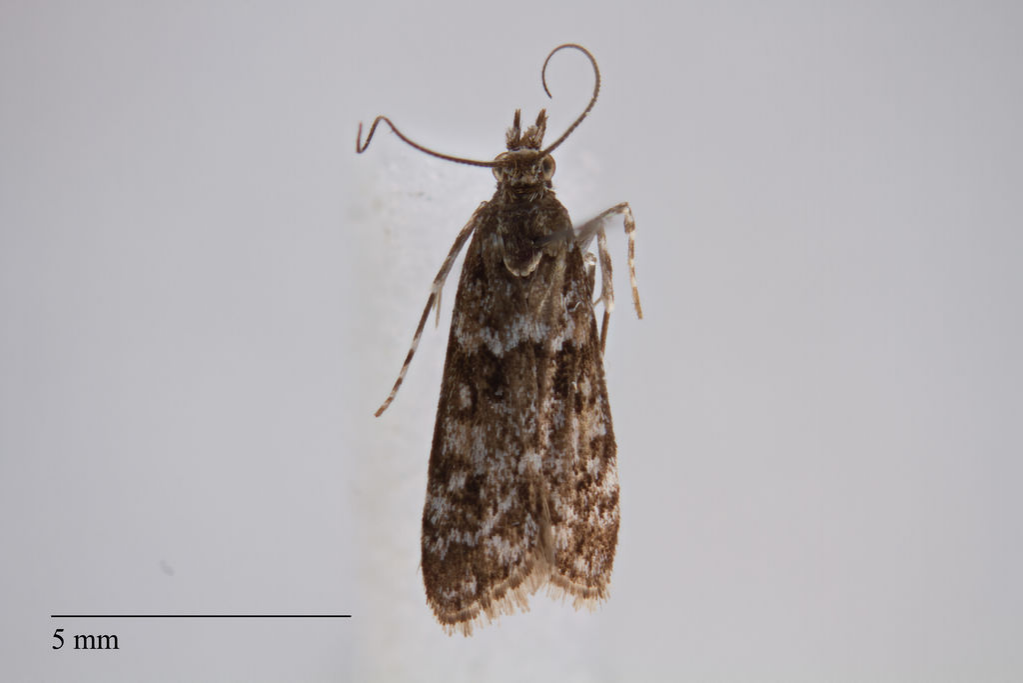
*Eudonia melanographa* (Hampson, 1907) from Teceira (Azores, Portugal) deposited in Coll. ZMUC (Credit: Anders Illum).

**Figure 4.**
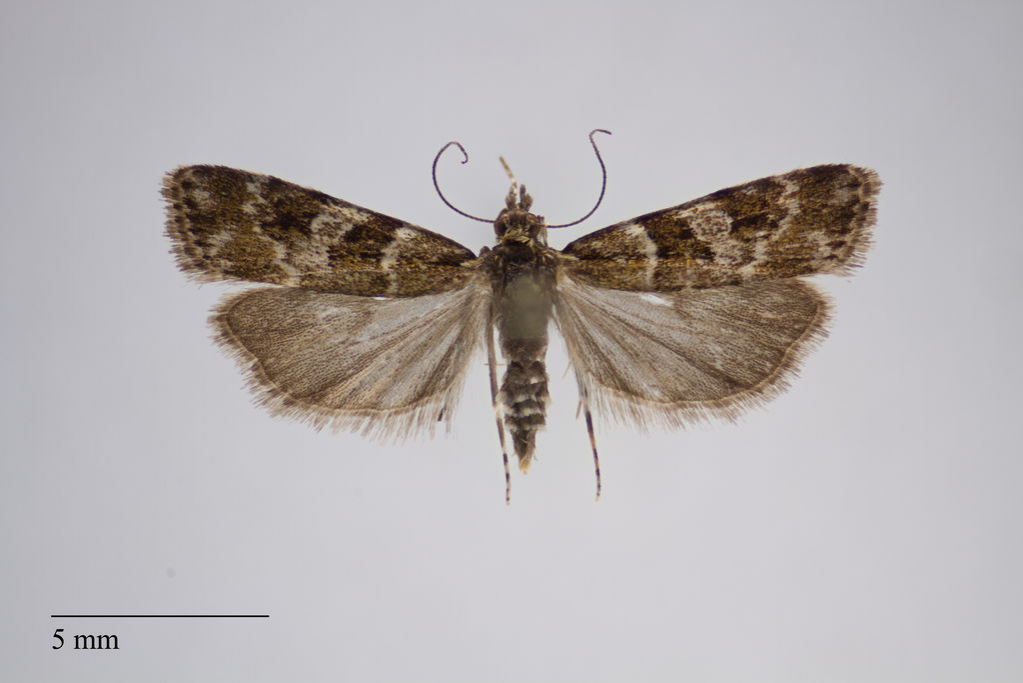
*Scoparia aequipennalis* Warren, 1905 from Pico (Azores, Portugal) deposited in Coll. ZMUC (Credit: Anders Illum).

**Figure 5.**
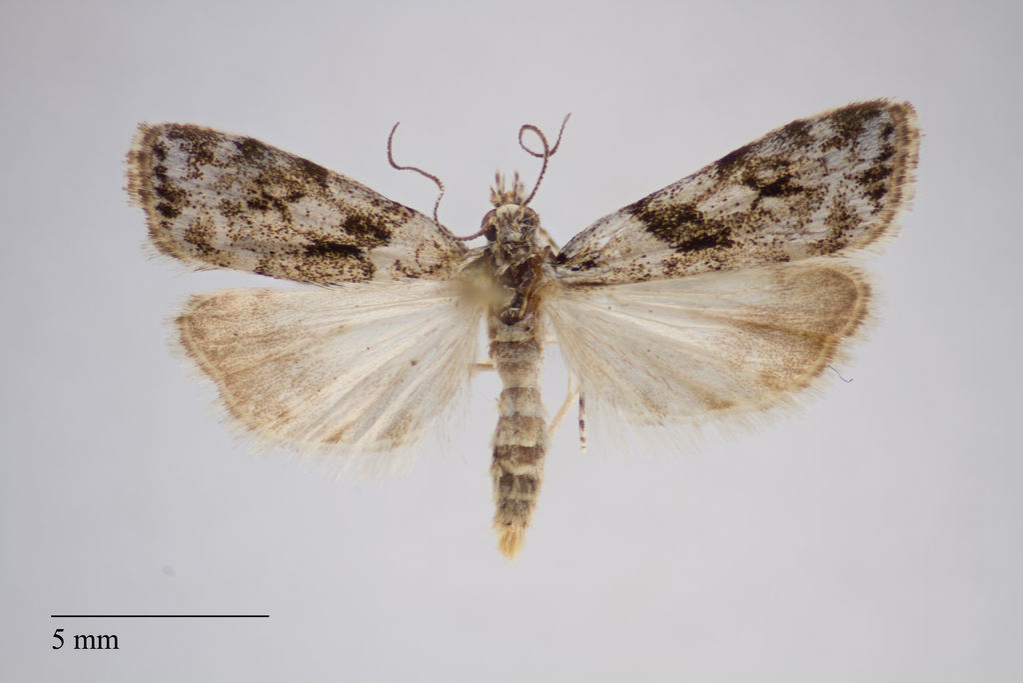
*Scoparia carvalhoi* Nuss, Karsholt & Meyer, 1997 paratype from Pico (Azores, Portugal) deposited in Coll. ZMUC (Credit: Anders Illum).

**Figure 6.**
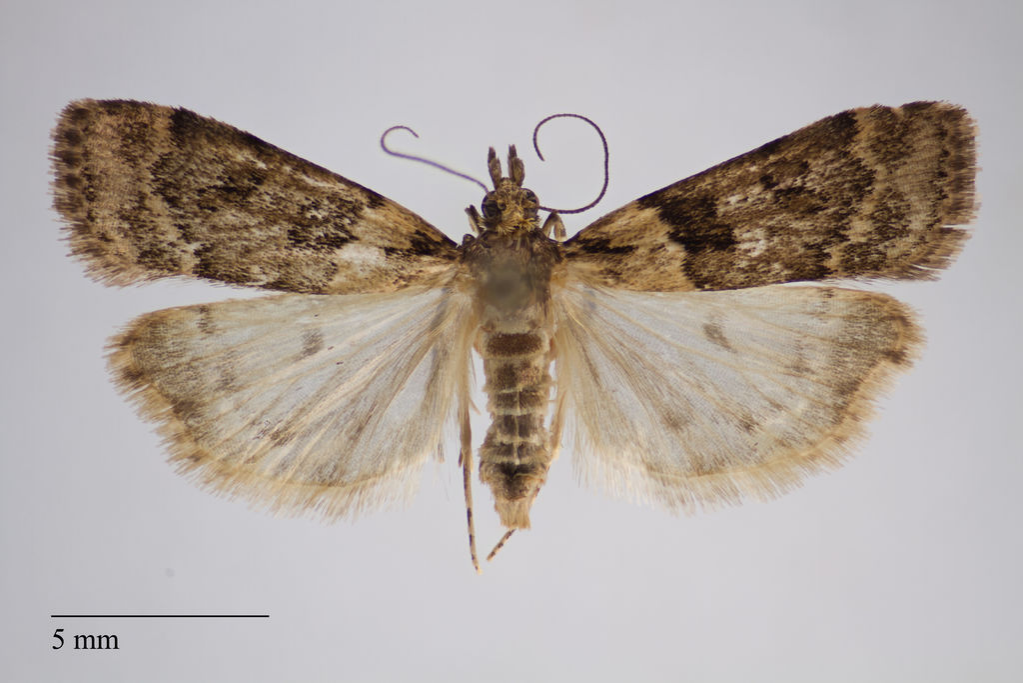
*Scoparia coecimaculalis* Warren, 1905 from Pico (Azores, Portugal) deposited in Coll. ZMUC (Credit: Anders Illum).

**Figure 7.**
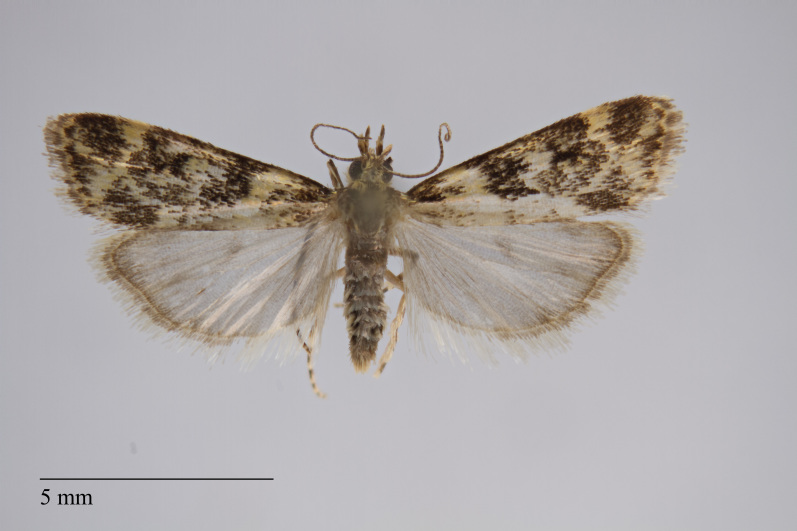
*Scoparia semiamplalis* Warren, 1905 from Pico (Azores, Portugal) deposited in Coll. ZMUC (Credit: Anders Illum).

**Figure 8.**
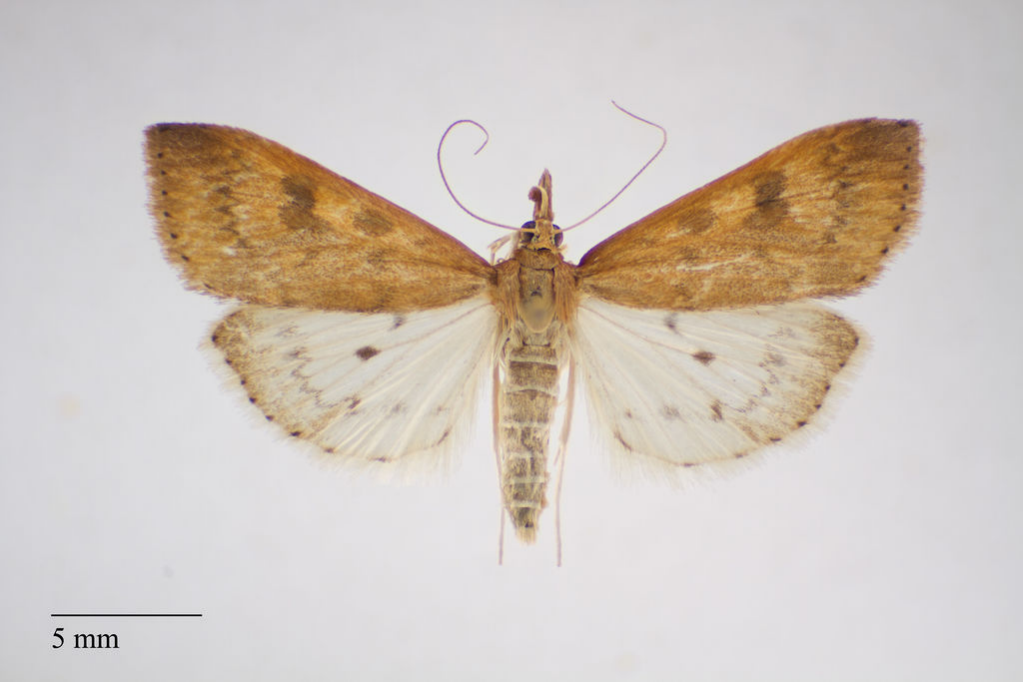
*Udea azorensis* Meyer, Nuss & Speidel, 1997from Pico (Azores, Portugal) deposited in Coll. ZMUC (Credit: Anders Illum).

**Figure 9.**
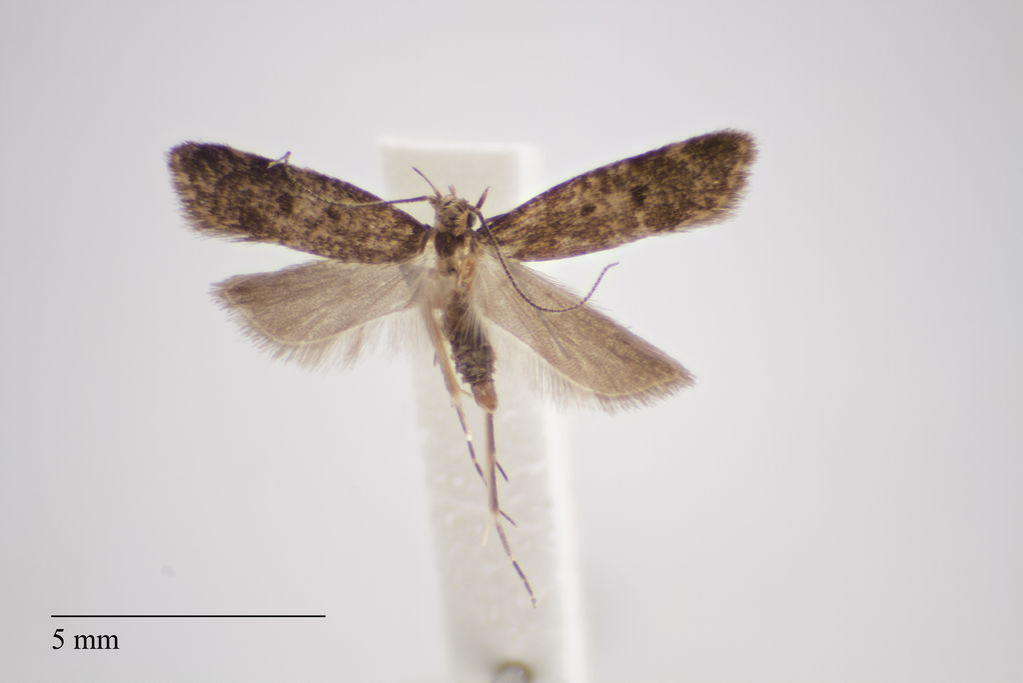
*Brachmia infuscatella* Rebel, 1940 from Santa Maria (Azores, Portugal) deposited in Coll. ZMUC (Credit: Anders Illum).

**Figure 10.**
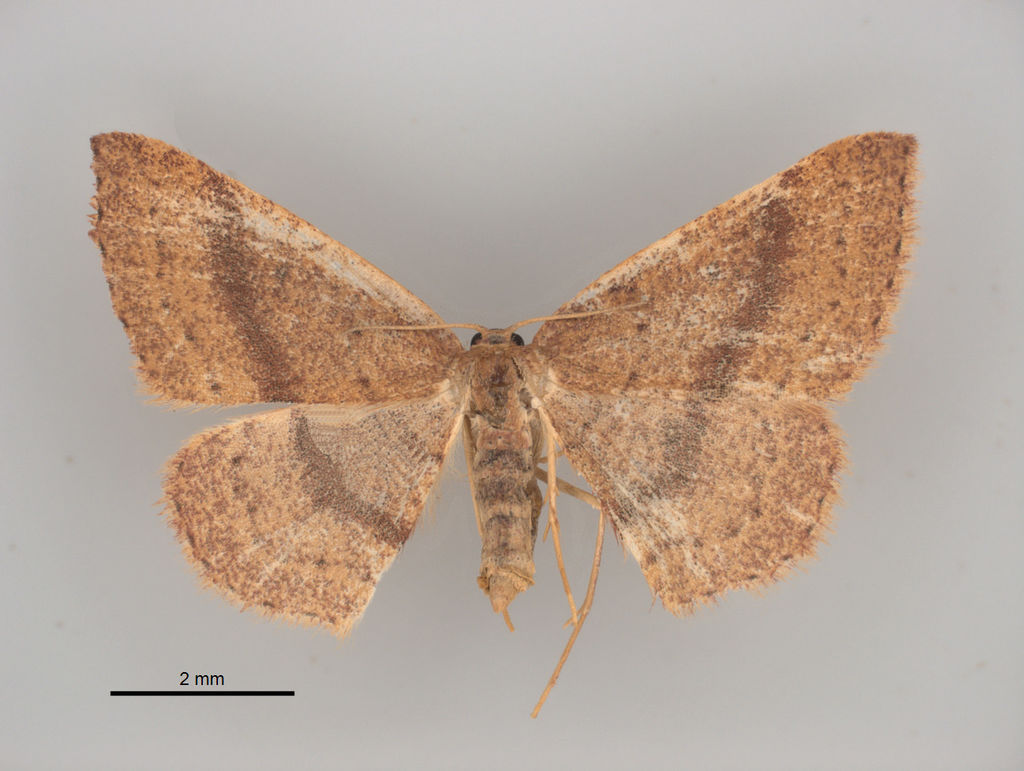
*Cyclophora azorensis* (Prout, 1920) from Mistérios Negros at Terceira (Azores, Portugal) (Credit: José V. Pérez Santa-Rita).

**Figure 11.**
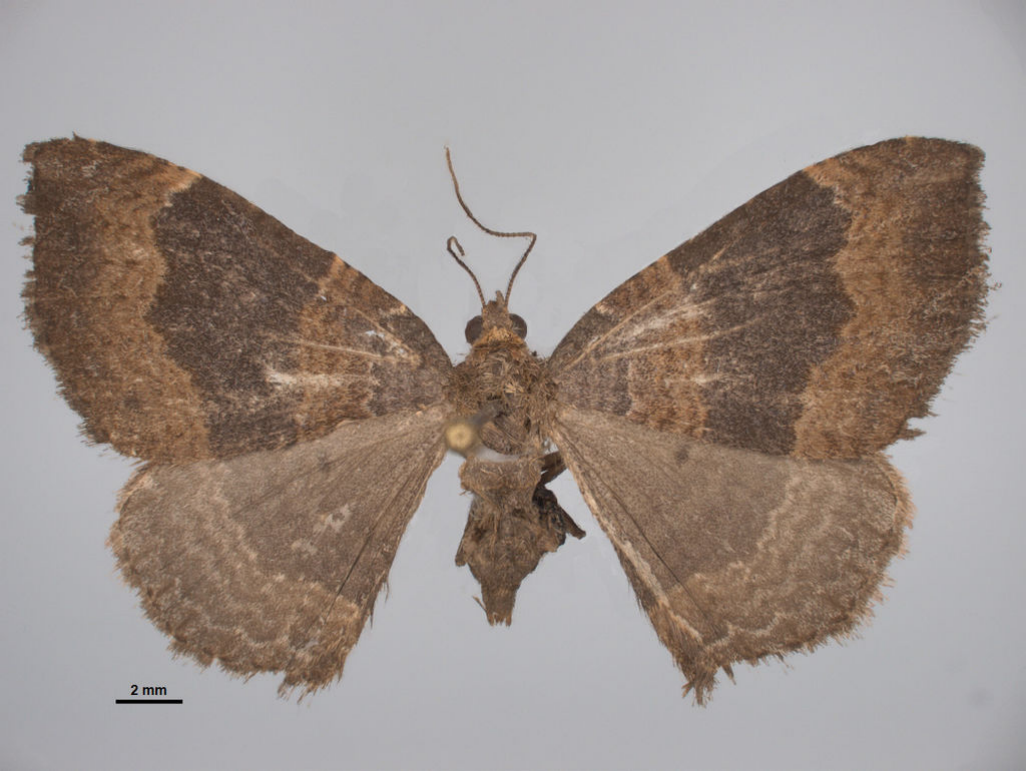
*Xanthorhoe inaequata* Warren, 1905, from Mistérios Negros at Terceira (Azores, Portugal) (Credit: José V. Pérez Santa-Rita).

**Figure 12.**
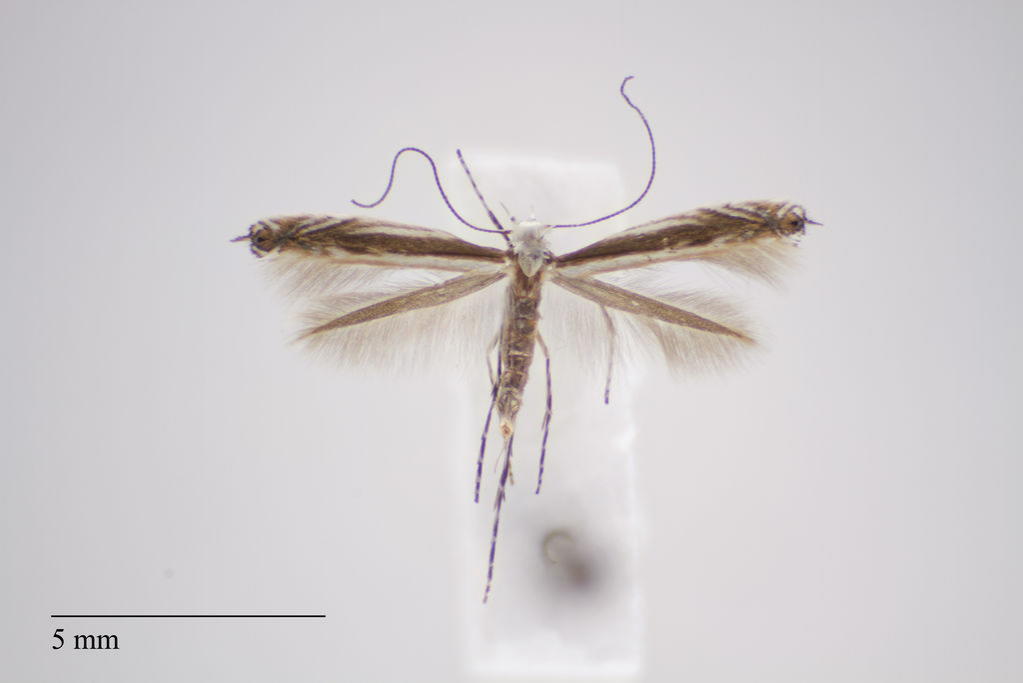
*Micrurapteryx bistrigella (Rebel, 1940) from Pico_* (Azores, Portugal) deposited in Coll. ZMUC (Credit: Anders Illum).

**Figure 14.**
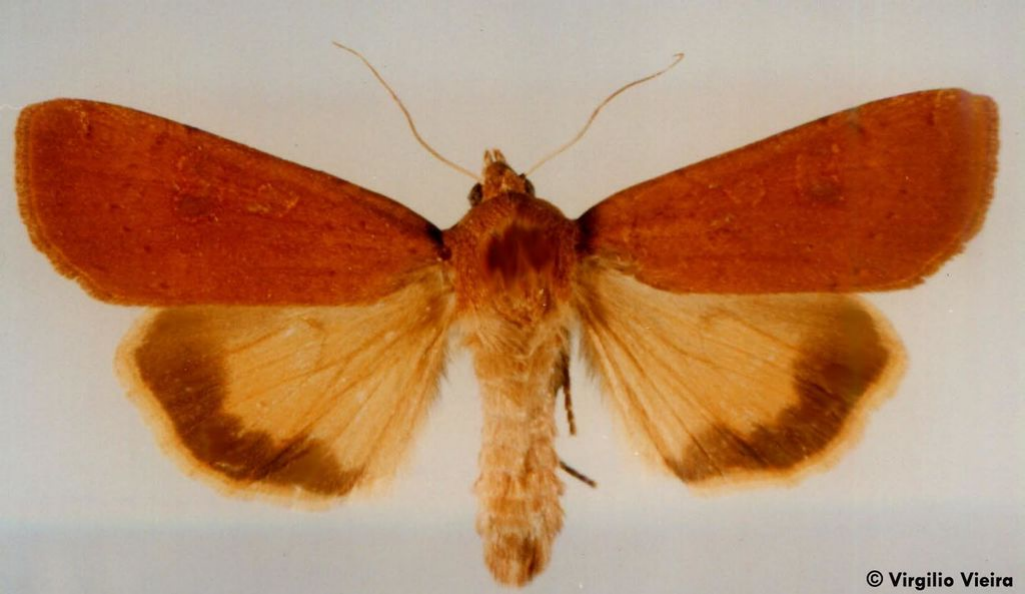
*Noctua atlantica* (Warren, 1905) from São Miguel (Azores, Portugal) (Credit: Virgílio Vieira).

**Figure 15.**
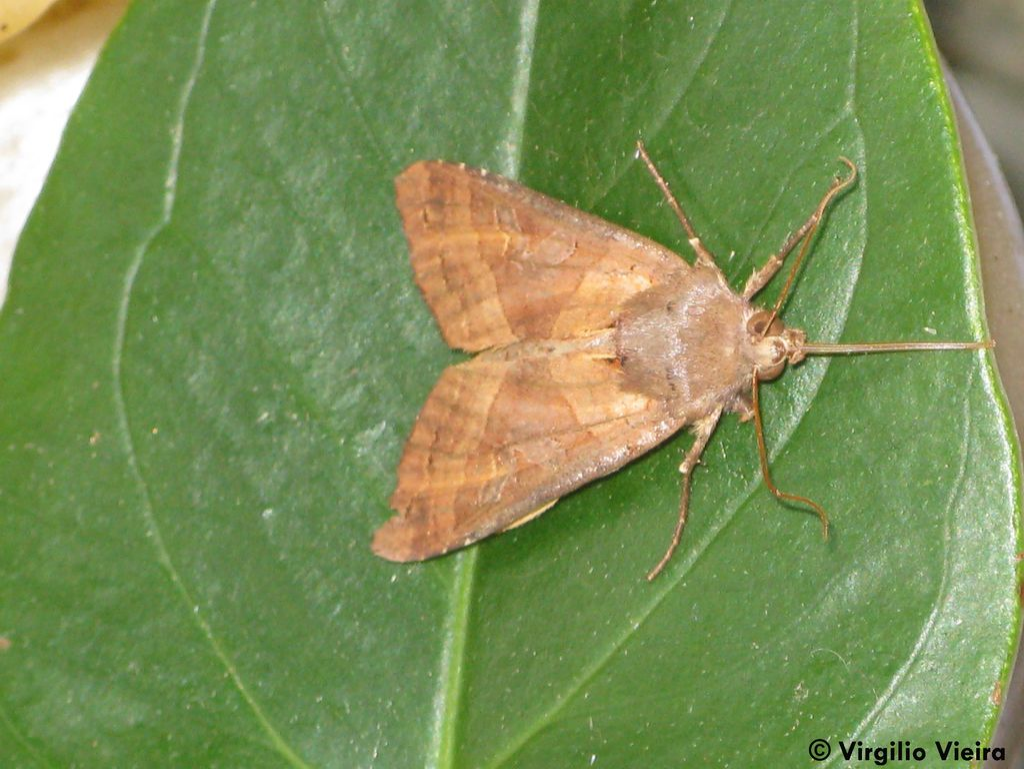
*Phlogophora interrupta (Warren, 1905)* from São Miguel (Azores, Portugal) (Credit: Virgílio Vieira).

**Figure 16.**
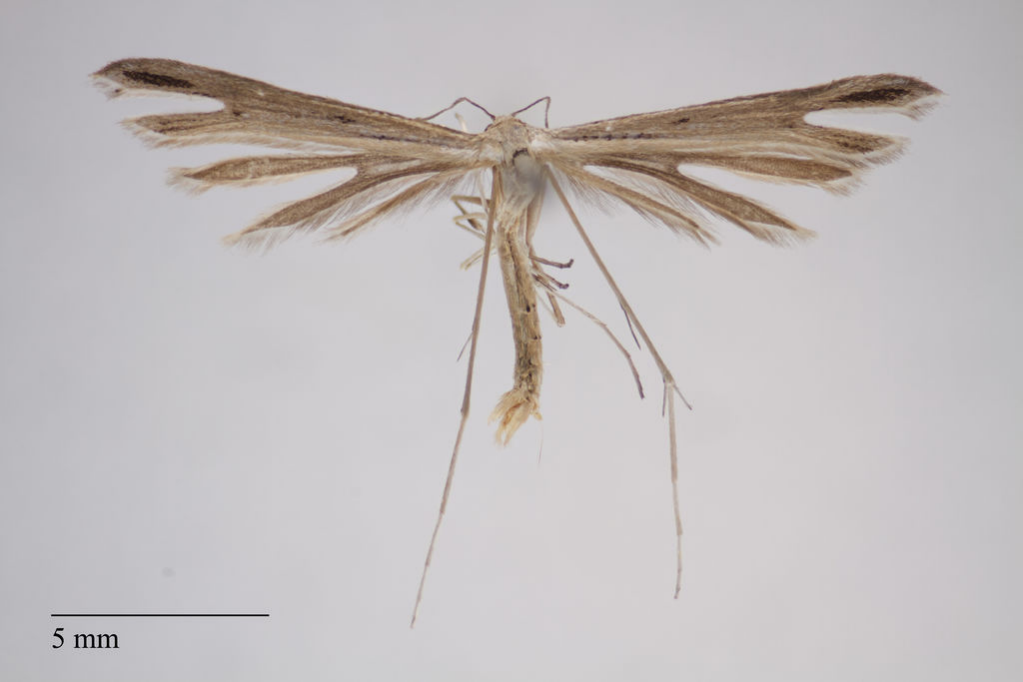
*Stenoptilia meyeri* Gielis, 1997 from São Miguel (Azores, Portugal) deposited in Coll. ZMUC (Credit: Anders Illum).

**Figure 17.**
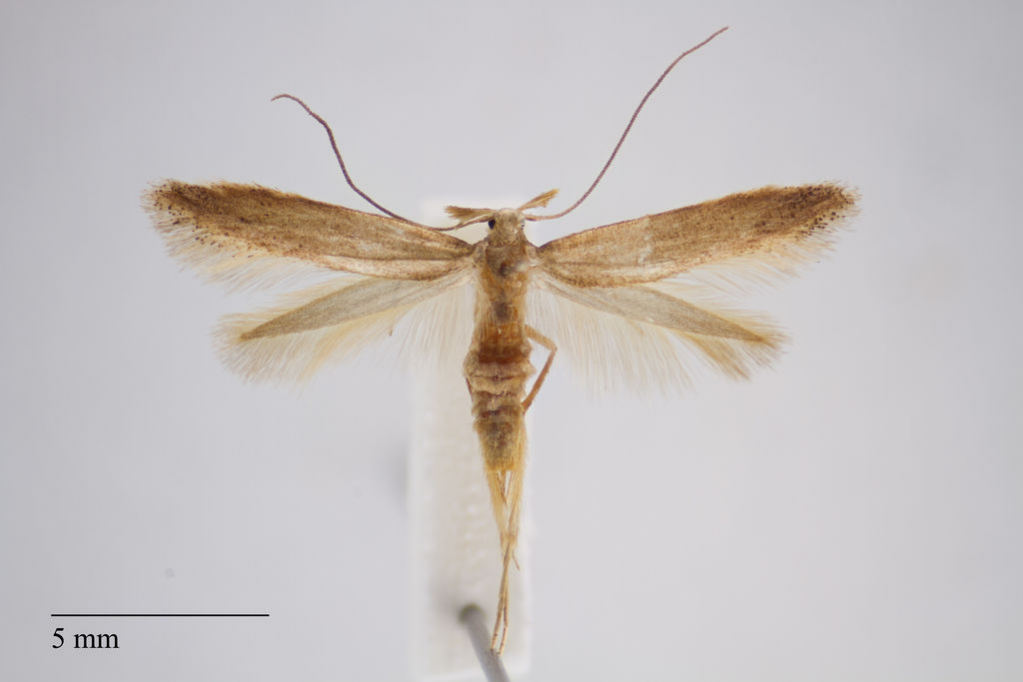
*Neomariania incertella* (Rebel, 1940) deposited in Coll. ZMUC (Credit: Anders Illum).

**Figure 18.**
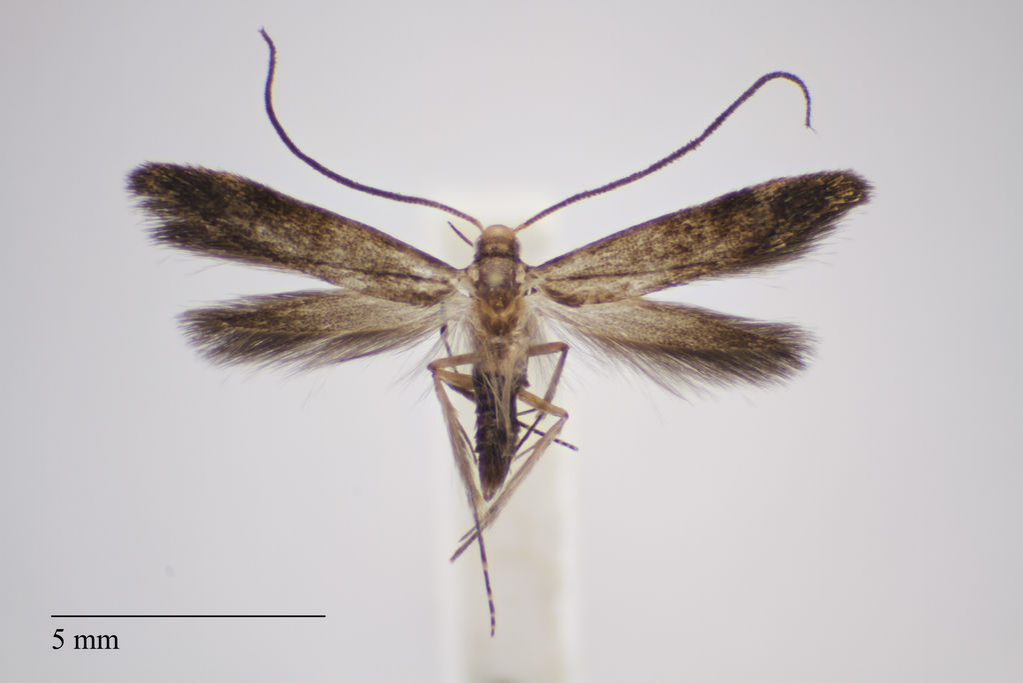
*Neomariania oecophorella* (Rebel, 1940) from São Miguel (Azores, Portugal) deposited in Coll. ZMUC (Credit: Anders Illum).

**Figure 19.**
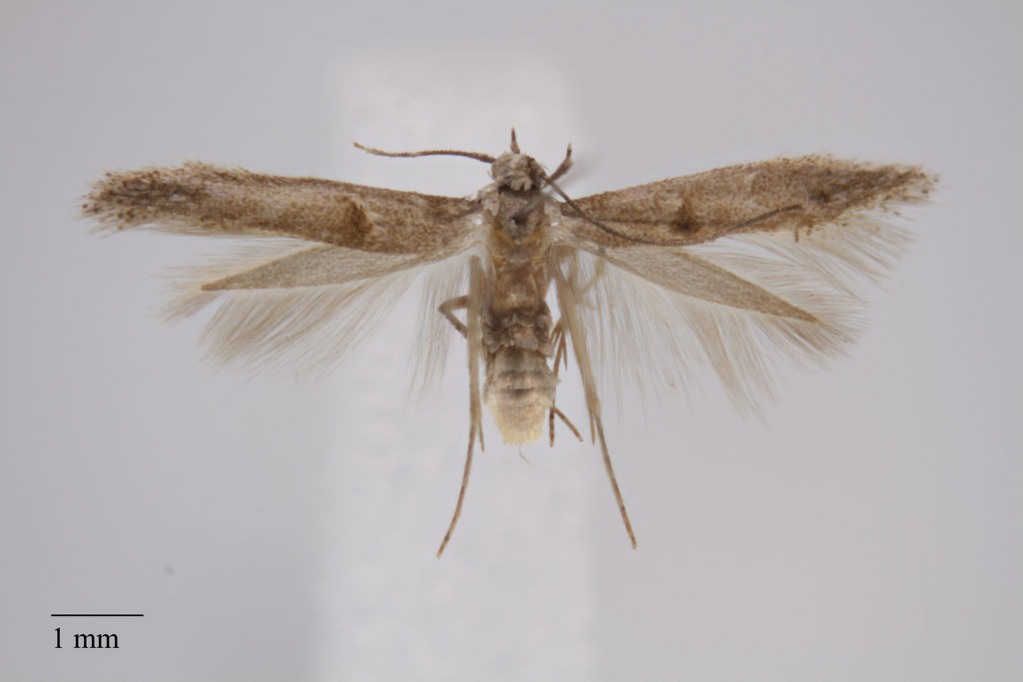
*Neomariania scriptella* (Rebel, 1940) from Azores (Portugal) deposited in Coll. ZMUC (Credit: Anders Illum).

**Figure 20.**
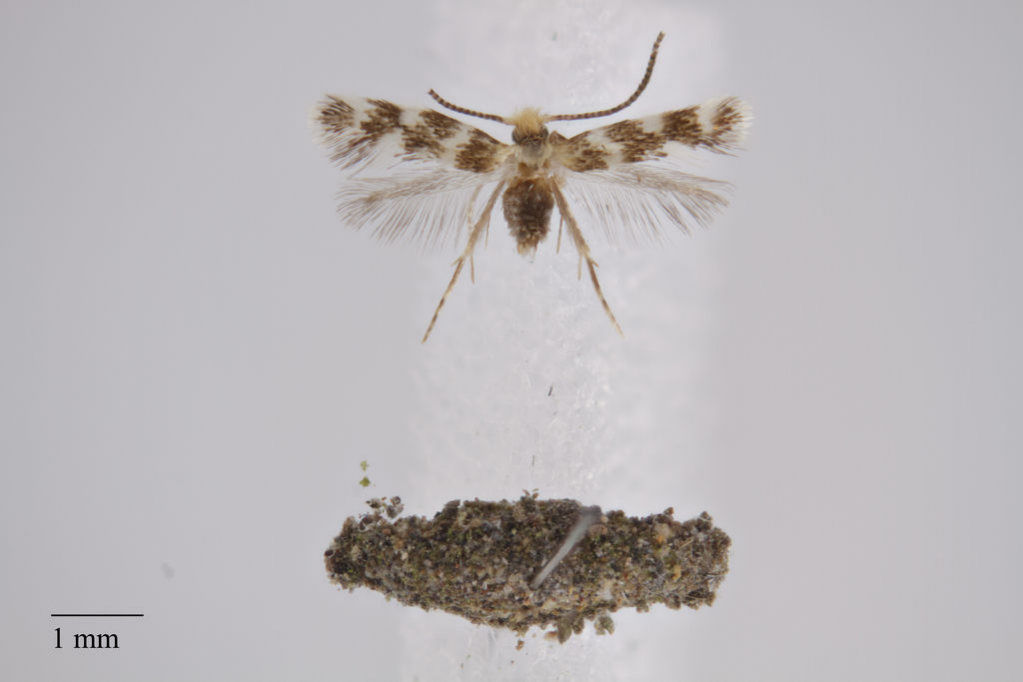
*Eudarcia atlantica* Henderickx, 1995 from São Miguel (Azores, Portugal) deposited in Coll. ZMUC (Credit: Anders Illum).

**Figure 21.**
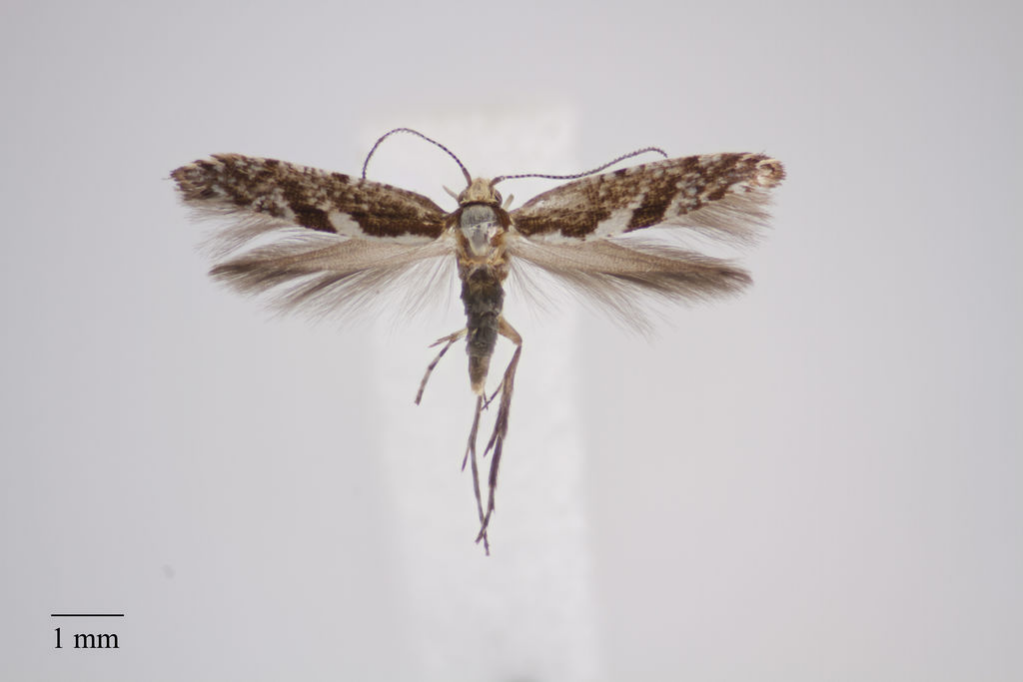
*Argyresthia atlanticella* Rebel 1940 from São Miguel (Azores, Portugal) deposited in Coll. ZMUC (Credit: Anders Illum).

**Figure 22.**
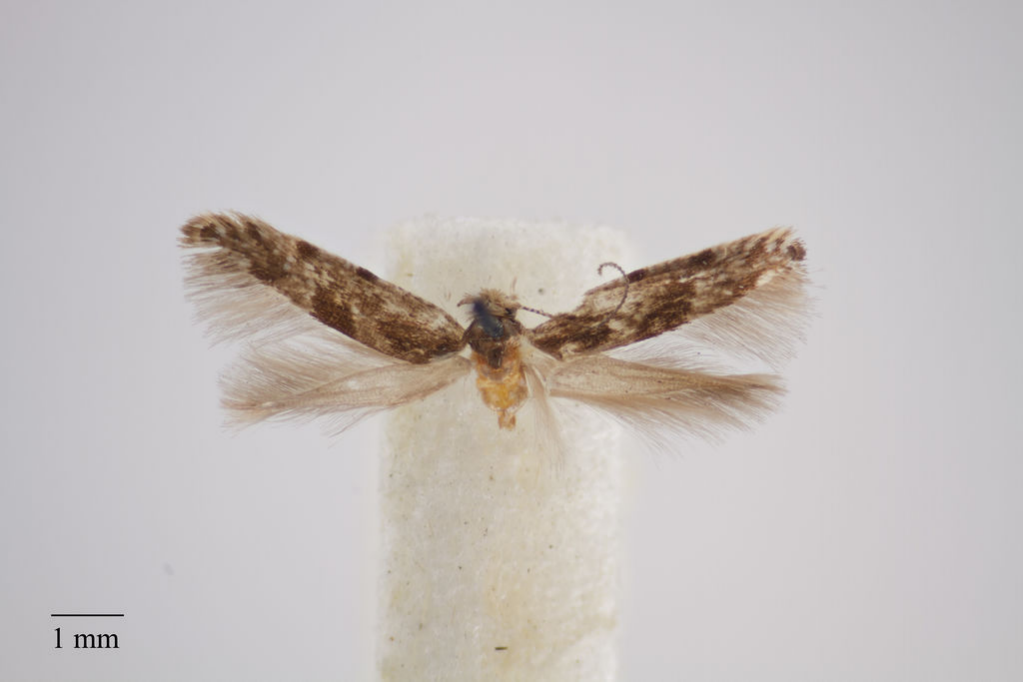
*Argyresthia poecilella* (Rebel 1940) type from São Miguel (Azores, Portugal) deposited in Coll. MZH (Credit: Anders Illum).

## Hosting institution

cE3c – Centre for Ecology, Evolution and Environmental Changes / Azorean Biodiversity Group and Universidade dos Açores, Dep. de Ciências e Engenharia do Ambiente, Angra do Heroísmo, Açores, Portugal
